# Trilogy-Constrained Acetabular Component for Recurrent Dislocation

**DOI:** 10.1155/2013/629201

**Published:** 2013-01-10

**Authors:** Annette Vest Andersen, Anne Grete Kjersgaard, Søren Solgaard

**Affiliations:** Department of Orthopedic Surgery, Gentofte Hospital, Niels Andersens Vej 65, 2900 Hellerup, Denmark

## Abstract

32 patients received a Trilogy- or Trilogy-Longevity-constrained acetabular liner for recurrent dislocations after total hip replacement. The constrained liner was inserted into a well-fixed Trilogy acetabular shell with snap fit. At 1.8-year followup (range 3–63 months), 4 patients had suffered further dislocation(s) (12%), and one patient had revision surgery for a loosened acetabular shell. Radiologic evaluation detected no definitively loose components, but one patient with progressing radiolucent lines around the femoral component and one patient with an acetabular cyst were found, as well as a patient with a loose locking ring (but otherwise no failure). The nineteen patients who were available for the present followup had a mean Harris Hip Score of 81. The constrained liner is an effective method of dealing with recurrent dislocations in well-fixed components.

## 1. Introduction

Dislocation remains one of the most common complications after primary and especially revision hip arthroplasty. The rate of dislocation is influenced by many different factors and ranges between 0.3 and 10% in primary arthroplasty [1–5] and between 4 and 28% after revision arthroplasty [1–4]. The incidence varies greatly in different studies with a much higher risk for patients with neuromuscular disease or lack of compliance resulting from dementia or substance abuse [1, 2]. However, the rate of recurring dislocations has also been associated with surgical approach (including soft tissue repair) [6], surgical volume [4], and choice of implant [2]. 

Many different methods have been used to solve the problem, both nonoperative and surgical methods. Nonoperative in the form of different kinds of immobilizing devices. Surgically by repositioning malpositioned components, inserting jumbo or bipolar heads, or longer necks and last but not least by using a constrained liner [1–3].

None of the methods mentioned above have been without complications. The use of different kinds/brands of constrained liners has been reported with mixed results. We report on the use of the Trilogy constrained liner (Trilogy and Trilogy Longevity) in a consecutive series of patients operated on because of recurrent dislocations.

## 2. Materials and Methods

We performed a retrospective review of all patients treated with a Trilogy constrained liner in the Hip Clinic Hørsholm Hospital, Denmark, in the period 2005–2009. The cohort comprised 38 patients all treated with a constrained acetabular insert because of recurrent dislocations (average 4.6 dislocations; range (1–10)), one was treated twice. The patients were identified by searching the electronic database for all revision hip arthroplasties. All these patients were reviewed and the ones who were revised using a constrained liner were included in the present study.

**Figure 1:**
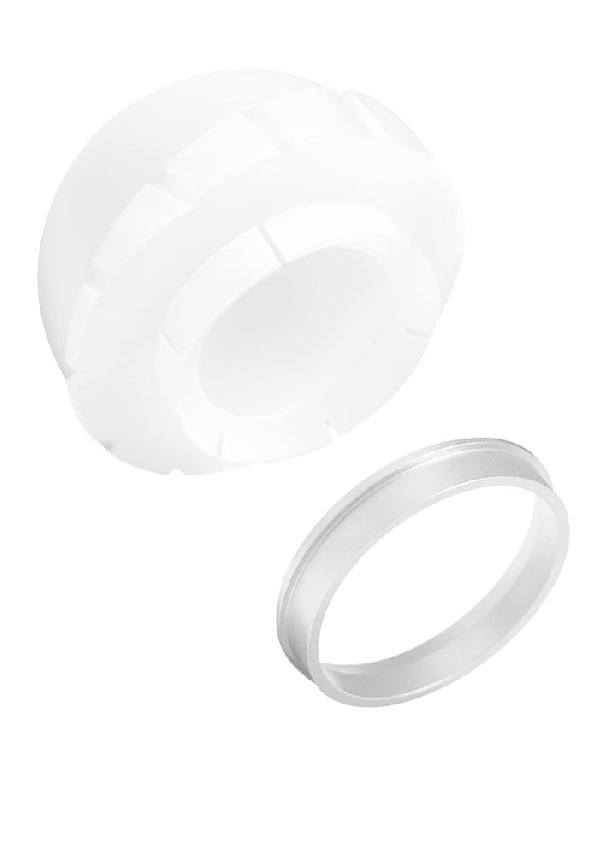
Trilogy constrained liner.

**Figure 2:**
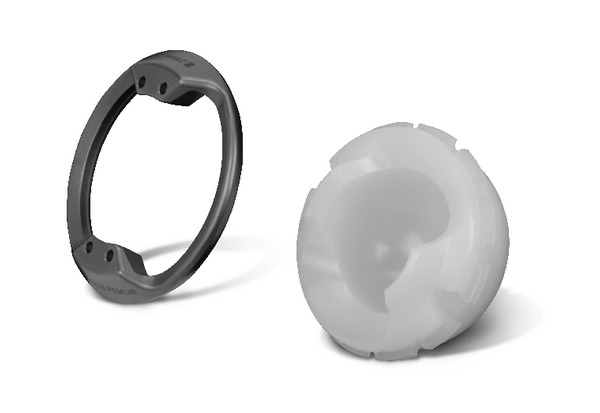
Trilogy Longevity constrained liner.

Of these patients, a number had to be excluded. Five patients were fitted with another brand of constrained liner and one emigrated and was lost to followup. That left 32 patients all with Bimetric femoral components and the Trilogy acetabular cup. The constrained liners used were either Trilogy (TC) (21 hips) or Trilogy Longevity (TL) (12 hips) (see Figures 1 and 2).

The 32 patients included 24 females and 8 males, 20 right hips (1 hip counts twice), and 13 left hips. The average age at constrained liner insertion was 74 years (range 46–86).

The primary diagnosis in the majority of cases was arthrosis (88%); the rest had avascular necrosis following internal fixation of a femoral neck fracture (12%). (for data on the groups divided by type of liner, see Table 1).

In addition to the hip problems, 3 patients suffered from dementia and 2 had an ongoing problem with alcohol abuse. No patients with neuromuscular disease were registered (5 patients were undetermined regarding the above-mentioned problems).

The patients were all operated through the posterolateral approach by one of the department's 4 senior surgeons. During surgery both stem and cup were tested. In all but one patient, both cup and stem were found to be well fixed. One patient had a loose stem, which was revised during the same procedure. The patients were mobilized using standard precautions (no adduction, no inward rotation, and no flexion past 90 degrees for the first 3 months).

At the time of followup, 7 patients had died. Three further patients had had additional surgery and had their constrained liner removed for different reasons (they were included in the study but not seen at followup). The rest of the patients were invited to a clinical examination including radiographs of the relevant hip. The radiographs were evaluated for loosening defined as migration of the components or progressive radiolucent lines. 

The average followup was 26 and 15 months for the Trilogy (range 2–63) and Trilogy longevity (range 4–26), respectively. Of the patients still alive, 3 were not seen for followup due to other health issues and personal obligations. The first two patients were interviewed over the phone; the third patient was not available for interviewing.

Twenty-three patients had only one previous surgical procedure (THA), 8 had been operated twice, and a single patient had 3 previous procedures. In addition to the primary THA's, the previous procedures were osteosynthesis of a femoral neck fracture (13%), revisions to treat dislocations (repositioning of cup and lengthening of the neck) (13%), one periprosthetic fracture (3%), one revision because of infection (3%), and one patient had the constrained liner replaced because of failure and another constrained liner inserted (3%) (For data on the groups divided by type of liner see Tables 2 and 3).

## 3. Results

Four patients had suffered from one to four further dislocations (12%), three patients with a Trilogy constrained liner, and 1 patient with a Trilogy longevity constrained liner. Two of these were also debrided due to deep infection. One patient had the acetabular shell revised because of loosening (TC), and 2 other patients had debridement with head and liner change because of infection.

Thus a total of 7 (21%) patients had 15 additional procedures including closed reductions. 

An additional patient has a loose locking ring but has not had any dislocations.

The time from insertion of the constrained liner to dislocation averaged 24 months.

18 patients were seen for the followup including radiographs and one only for additional radiographs. No migration was seen in any patients; one patient had a slight progression of radiolucent lines at the femoral component. One patient had a small cyst in the lateral part of the acetabulum suggesting osteolysis.

The patients who for various reasons were not seen for the present followup had been seen previously 2–15 months postoperatively. Apart from the already noted failures no migration or progression of radiolucent lines was found. The patients that were seen for followup had an average Harris Hip Score (HHS) of 81.

In the group of patients who suffered further dislocations despite the constrained liner, one suffered from dementia and one had a problem with alcohol abuse.

No difference between the TC liner and the TL in any of parameters investigated could be demonstrated.

## 4. Discussion

The number of different constrained liners on the market is abundant: bipolar as well as tripolar [7, 8] (to match the even larger number of different shells, head, and stem types). Published data is currently only available for a few of the constrained liners: Osteonics and Omnifit (Osteonics Corp./Stryker Howmedica) [9–21], Trident (Stryker) [22, 23], S-ROM (DePuy) [24–26], Duraloc (DePuy) [27, 28], Ringloc (Biomet) [20, 29], and Trilogy (Zimmer) [20, 28, 29]. Some of the studies include patients with more than one brand of liner [20, 24, 25, 28, 29].

In addition to different kinds of liners, the indication for insertion of a constrained liner differs as well. We inserted constrained liners as a measure to prevent further dislocations in patients with repeated dislocations, and the constrained liner has not been used in primary surgery in the present series. Some of the published papers deal with a similar material [13, 14, 17, 22, 29], the rest of the published data are on a more diverse group of patients with indications varying from repeated dislocations or intraoperative instability (both primary procedures and revisions) to neurologic impairment and revision procedures on patients with Girdlestone status [9–11, 15, 16, 18–21, 23–25, 27, 28]. 

As the type of constrained insert (as well as how it is inserted), indication, surgical approach, number of patients, and follow-up time are widely different, so are the results. Shrader et al. (110 hips) [15], McCarthy and Lee (39 hips), [19] and Stanton et al. (13 hips) [25] had no redislocations at followup after 3.2, 4.6, and 3.5 years, respectively, although 2 cases of subluxation (2%) have been noted [15]. Anderson et al. (21 hips) [26], Pattyn et al. (46 hips) [20], and Berend et al. (755 hips) [24] published a dislocation rate of 29%, 21.7%, and 17.5% at a followup of 2.5, 2.4, and 10.7 years, respectively. All these studies included a patient group where the indication was “mixed.”

Looking at studies of patients with recurring dislocations (including dissociations), the success rate also varies. Goetz et al. (56 hips) [14], Shapiro et al. (85 hips) [17], Khan et al. (34 hips) [22], and Knudsen et al. (40 hips) [29] found redislocation rates of 5.3%, 2.3%, 2.9%, and 7.5% with a followup between 2 and 7 years. The redislocation rate in our material was 12%.

Equally important is the rate of “failure” of the constrained liner. This includes not only the dislocations and dissociations, and the breakage of different parts of the liner, but also shells that are pulled out because of increased stress, and revisions because of loosening of the acetabular shell. Failure rates of 5.3%, 5.8%, 14.7%, and 10% have been described [14, 17, 22, 29]. In our material in addition to the dislocations, one locking ring failed (without dislocation), and one acetabular shell was loose. No other failures were registered, probably because the constrained liner only had been used in well-fixed acetabular shells.

The rate of redislocation was 12% and the total failure rate (including loosening of the acetabular shell and loosening of the ring) was 18%.

## 5. Conclusion

The constrained liner has in the present material been used only as a salvage procedure in a population of patients with recurrent dislocations, which might explain the rather high rate of failure. Furthermore, patients with conditions predisposing to hip-dislocation also seemed to be at a higher risk for subsequent failure of the constrained liner. 

However, 88% of the patients had no further dislocations or loosening of the implant, and no better solution in this group of patients seems available at present.

We continue to use this method as a salvage measure for patients with recurring dislocations and well-positioned and well-fixed components.

## Figures and Tables

**Table 1:** Groups divided by type of liner.

	Trilogy (TC)	Trilogy longevity (TL)
Number of patients	21	12
Male	7 (33%)	1 (8%)
Female	14 (67%)	11 (92%)
Mean age (time of insertion)	73 years	75 years
Cemented stem	10 (48%)	3 (25%)
Uncemented stem	11 (52%)	9 (75%)

**Table 2:** Number of previous procedures.

	Trilogy	Trilogy longevity
1	15 (72%)	8 (67%)
2	5 (23%)	4 (33%)
3	1 (5%)	0 (0%)

**Table 3:** Indications for previous procedures.

	Trilogy	Trilogy longevity
Arthrosis	18 (86%)	11 (92%)
Osteosynthesis femoral neckfracture	3 (14%)	1 (8%)
Avascular necrosis following femoral neck fracture	3 (14%)	1 (8%)
Infection	1 (5%)	0 (0%)
Failure of constrained liner	0 (0%)	1 (8%)
Reoperation to treat dislocations	2 (10%)	2 (17%)
Periprosthetic fracture	1 (5%)	0 (0%)
